# ﻿Molecular and morphological evidence reveals a new fern species of *Hymenasplenium* (Aspleniaceae) from south and southwestern China

**DOI:** 10.3897/phytokeys.211.90363

**Published:** 2022-10-20

**Authors:** Yanfen Chang, Guocheng Zhang, Zhixin Wang, Limin Cao

**Affiliations:** 1 College of Life Sciences, Hengyang Normal University, Hengyang 421008, Hunan, China Hengyang Normal University Hunan China; 2 Hunan Key Laboratory for Conservation and Utilization of Biological Resources in the Nanyue Mountainous Region, Hunan, China Hunan Key Laboratory for Conservation and Utilization of Biological Resources Hunan China

**Keywords:** *Hymenasplenium*, *H.excisum* subclade, new taxon, species complex, taxonomy

## Abstract

*Hymenaspleniumobtusidentatum*, a new fern species of the *H.excisum* subclade of *Hymenasplenium* (Aspleniaceae) from south and southwestern China was described. Molecular phylogenetic analyses and morphological observations of *H.obtusidentatum* and related species clearly indicated that this is a distinct taxonomic entity. Phylogenetically, *H.obtusidentatum* was confirmed to represent a diverging lineage in the *H.excisum* subclade of *Hymenasplenium* and was closely related to one lineage that includes accessions identified as *H.obscurum*, *H.pseudobscurum* and *H.tholiformis*. Morphologically, *H.obtusidentatum* can be distinguished by the combination of its lamina base truncate, stipe not shiny and with color of reddish brown to dark brown, and pinna marginal teeth that are not sharp, but blunt or rounded. A complete species description and comparison with related species in the *H.excisum* subclade were provided. The holotype of *H.obtusidentatum* was designated.

## ﻿Introduction

*Hymenasplenium* Hayata (1927: 712) is one of the two genera in the nearly globally distributed and species-rich fern family Aspleniaceae (Murakami 1995; Murakami et al.1999; Schneider et al. 2004; PPG I 2016; Xu et al. 2018a, 2020). Compared with the large genus *Asplenium* L., which is estimated to contain more than 700 species (PPG I 2016; Xu et al. 2020), *Hymenasplenium* consists of only about 60 species (Xu et al. 2018a). Three major clades and several subclades have been recognized in recent phylogenetic studies of this genus (Chang et al. 2018; Xu et al. 2018a). An exploration of species diversity and taxonomy within *Hymenasplenium* is still in progress because of the existence of numerous species complexes (Lin and Viane 2013) and extensive cryptic speciation in this genus (Ching 1965; Xu et al. 2018a; Zhang et al. 2021). Recently, our understanding of *Hymenasplenium* has increased due to the discovery of many new species and taxonomic research in some complex groups, such as *H.unilateral* s. l. and the *H.retusulum*-*H.latidens* groups (Chang et al. 2018; Xu et al. 2018b, c, d, e, 2019a, b; Zhang et al. 2021). However, many species in *Hymenasplenium* are still poorly understood and awaiting further study (Lin and Viane 2013; Chang et al. 2018; Xu et al. 2018a).

The *H.excisum* subclade belongs to one of the six subclades in the Old World clade of *Hymenasplenium* (Xu et al. 2018a). In this subclade, several distinct lineages have been discovered, but only four species have been described and widely accepted: *H.excisum* (C. Presl) S. Lindsay, *H.obscurum* (Blume) Tagawa, *H.pseudobscurum* Viane, and the recently published *H.tholiformis* Liang Zhang, W.B. Ju & K.W. Xu (Lin and Viane 2013; Xu et al. 2018a; Qiu et al. 2022). Xu et al. (2018a) estimated that at least six undescribed species exist in this subclade and numbered four of them from sp8 to sp11, but without taxonomic description and treatment. Of these four described species and undescribed taxa, *H.excisum* and *H.obscurum* were reported to have a large distribution area from south to southwestern China, and tropical Asian to tropical Africa, but the occurrence of the true *H.obscurum* in China has been doubted (Lin and Viane 2013). *Hymenaspleniumpseudobscurum* distributes in south and southwestern China, and parts of tropical Asia, such as northern Thailand and Vietnam. *Hymenaspleniumtholiformis* is found to be endemic to southeastern Xizang of China. The undescribed sp8 and sp9 have been found only in Yunnan province in southwestern China, and sp10 and sp11 have been found in Hainan province in southern China. This indicates that south to southwestern China is one of the diversity and distribution centers of the *H.excisum* subclade (Lin and Viane 2013). Thus, further exploration of the species diversity and speciation of this group in south and southwestern China is necessary.

As a continued effort to clarify the species diversity and taxonomy of *Hymenasplenium*, we conducted fieldworks in south and southwestern China. During these trips, we collected some specimens of a new taxon of *H.excisum* subclade that were obviously different from all other species of the subclade in both morphology and phylogeny analyses. To clarify its taxonomic status, we studied the morphological differences between this taxon and related species and investigated the distinction of this putative new species by analyzing chloroplast DNA sequences for members of the monophyletic paleotropical clade of *Hymenasplenium* (Murakami and Moran 1993; Murakami and Schaal 1994; Murakami 1995; Gabancho and Prada 2011; Xu et al. 2018a). Here, we present our results, provide a description of this new taxon, and compare it with related species in the *H.excisum* subclade.

## ﻿Materials and methods

### ﻿Morphological studies

The morphological characteristics of the new species were observed in the field. Herbarium specimens of *Hymenasplenium* at PE, KUN, HITBC and PYU were studied. Digital specimens of related species of the new taxon were examined from the online databases of CVH (https://www.cvh.ac.cn/) and JSTOR Global Plants (https://plants.jstor.org/).

### ﻿Spore counting and imaging

Spores were obtained from newly collected specimens and examined with an Olympus BX-51 light microscope to examine aborted spores and determine the number of spores per sporangium. Mature sporangia from each specimen were removed and ruptured with a needle tip. The number of spores per sporangium was counted. The presence of 64 spores per sporangium was considered an indicator of the absence of apomictic reproduction (Wagner and Chen 1965; Dyer et al. 2012). Spore ornamentation was examined using a tabletop scanning electron microscope (ZEISS EVO LS 10) with spores sputter-coated with gold particles.

### ﻿Chloroplast DNA sequencing and phylogenetic analyses

To clarify the phylogenetic position of the new species, the taxa were sampled to include representatives of all the six subclades in the Old World clade of *Hymenasplenium* (Xu et al. 2018a). DNA sequences of plastid markers of 72 accessions representing 29 species of *Hymenasplenium* were sampled. Three species of the New World clades were used as outgroups. Voucher information and GenBank accession numbers for each sampled taxon were provided in Appendix 1.

Genomic DNA was extracted from the silica gel-dried leaf material of each sampled individual using the modified CTAB method (Doyle and Doyle 1987). Three regions of the chloroplast genome were amplified and sequenced using established primers and protocols, including the *rbcL* gene (Taberlet et al. 1991), *rps4-trnS* region (partial *rps4* gene and *rps4-trnS* intergenic spacer) (Schneider et al. 2005), and *trnH-psbA* intergenic spacer (Ebihara et al. 2010). These chloroplast regions were selected because they have previously been successfully used to assess relationships in *Hymenasplenium* (Murakami et al. 1999; Schneider et al. 2004; Xu et al. 2018a). For each of the three chloroplast regions, identical sequences of specimens from the same location were reduced to a single exemplar sequence and deposited in GenBank (see Appendix 1 for accession numbers).

Sequences were edited using the Staden Package (Staden et al. 2000), automatically aligned with Clustal X (Thompson et al. 1997), and manually corrected in BioEdit v.7.0.1 (Hall 2004). Special attention was given to the detection of ambiguously aligned regions and putative sequencing errors. A combined *rbcL*, *rps4-trnS*, and *trnH-psbA* chloroplast alignment was constructed. The sequences of the three cpDNA fragments were combined because they were inherited together. Ambiguous indels were excluded, and unambiguous indels were coded and scored using GapCoder (Young and Healy 2003). The datasets comprising sequences from the three chloroplast regions were analyzed using maximum parsimony (**MP**), maximum likelihood (**ML**) and Bayesian inference (**BI**). Maximum parsimony analyses were carried out in PAUP* 4.0b10 (Swofford 2002) using the heuristic search mode, 1,000 random starting replicates, and TBR branch swapping, with MULTREES and Collapse on. Bootstrap values were estimated using 1,000 bootstrap replicates under the heuristic search mode, each with 100 random starting replicates. Maximum likelihood analyses were carried out in PhyML 3.0 (Guindon et al. 2010) using default settings, and the best fit models for the parameter-based analyses were selected using jModelTest (Posada 2008) with the Akaike information criterion (Akaike 1974). Parameter values were estimated simultaneously with the analyses. Bayesian inference was carried out in MrBayes 3.1.2 (Huelsenbeck and Ronquist 2001) with four chains and the model selected by jModelTest with the Akaike information criterion (Akaike 1974). Chains were run for two million generations, and trees were sampled every 100 generations. Convergence was evaluated by examining the standard deviation of split frequencies among runs and by plotting the log-likelihood values from each run using TRACER v.1.4 (Rambaut and Drummond 2007). The remaining trees were used to calculate a 50% majority-rule consensus topology and posterior probabilities (**PP**).

## ﻿Results

### ﻿Morphological comparison and spore counting

**Figure 1.**
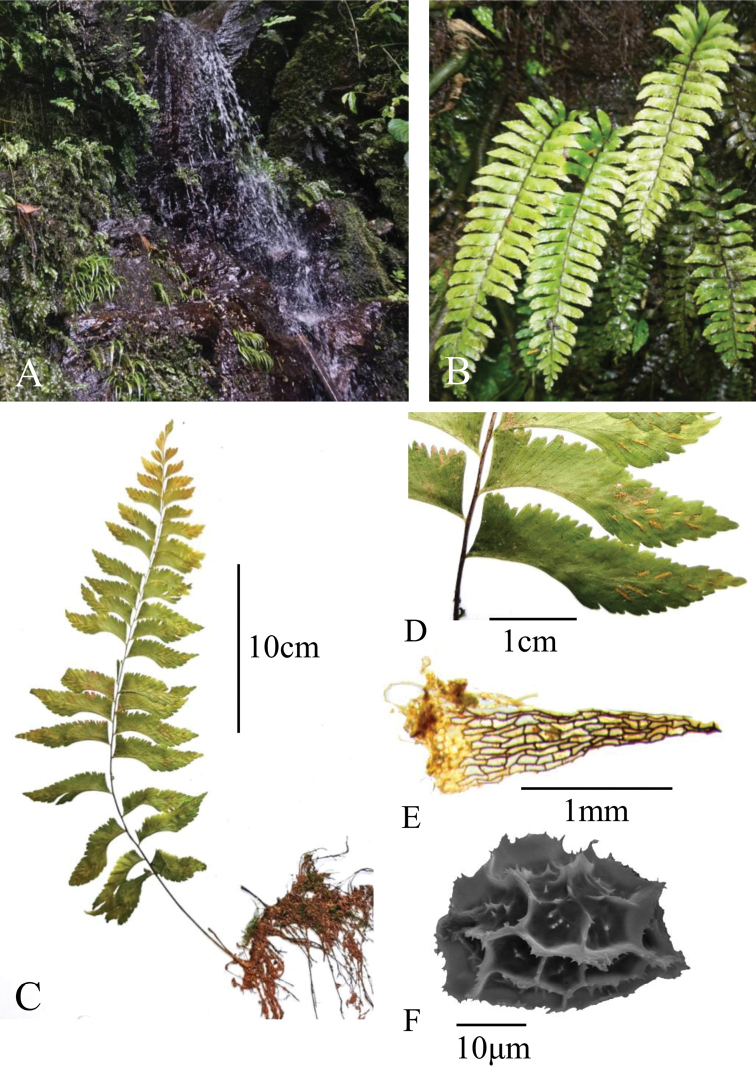
*Hymenaspleniumobtusidentatum***A** habit **B, C** plant **D** pinnae in middle part of leaf **E** scale **F** spore.

**Figure 2.**
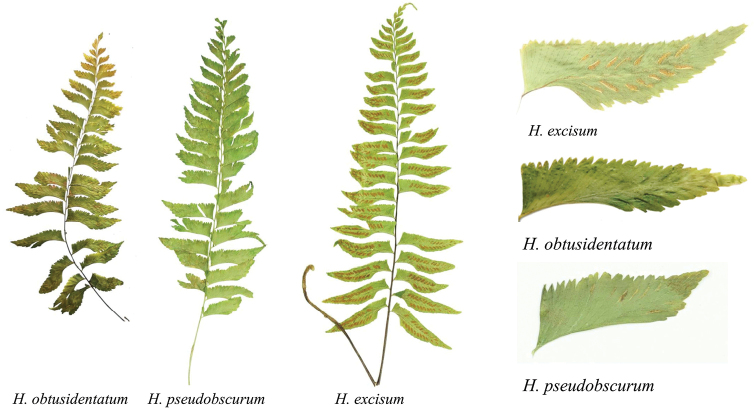
Comparison of frond sketches of representative specimens available (*H.obtusidentatum*: Chang1258, *H.pseudobscurum*: Chang1010, *H.excisum*: Chang992) for species of the *Hymenaspleniumexcisum* subclade.

Like most species in *Hymenasplenium*, the new species has a long-creeping rhizome, once-pinnate laminae, asymmetrical pinnae, and elliptic to reniform spores. However, the new species can be distinguished from other species in the genus by the combined characteristics of truncate lamina base, reddish brown to dark brown stipe and rachis, stipe not shiny and with scale or subglabrous, and pinna marginal teeth not sharp, but blunt or rounded (Figs 1, 2). In contrast, the closely related species *H.obscurum* and *H.pseudobscurum* have not shiny but dull green to grayish green stipe, whereas *H.excisum* and *H.tholiformis* have shiny and dark purple to black stipe. The comparison of morphological characteristics to differentiate the described species in the *H.excisum* subclade is shown in Table 1. The materials of the new taxon examined in this study contained 64 spores in each sporangium. Thus, *H.obtusidentatum* may not be an apogamous species.

**Table 1.** Comparison of morphological characters to differentiate the new taxon (*Hymenaspleniumobtusidentatum*) and the four described species in the *H.excisum* subclade.

Characters	*H.obtusidentatum*	*H.excisum*	*H.obscurum*	*H.pseudobscurum*	*H.tholiformis*
Size of lamina	15–30 × 8–15 cm	20–40 × 10–15 cm	20–30 × 5–10 cm	20–25 × 5–10 cm	13–16 × 3–5 cm
Lamina base	truncate	truncate and widest	truncate	truncate	truncate
Rhizome size	2–4 mm in diam.	3–5 mm in diam.	3–5 mm in diam.	3–5 mm in diam.	2 mm in diam.
Stipe color	not shiny, reddish brown to dark brown	shiny, dark purple to black	not shiny, dull green to grayish green	not shiny, dull green to grayish green	shiny, black purple
Pinna shape	trapeziform to falcate	trapeziform to falcate	trapeziform to falcate	trapeziform to falcate	trapeziform
Size of middle pinnae	3.5–8 × 1–1.8 cm	5–10 × 1.3–2 cm	3.5–7 × 0.8–1.3 cm	2.5–4 × 0.8–1.8 cm	2.5–8 × 0.6–1 cm
Shape of pinna apex	acute or rarely obtuse	acute to obtuse	obtuse to subacute	obtuse to subacute	round
Pinnae marginal teeth	blunt or rounded	sharp	sharp to blunt	sharp to blunt	sharp
Number of basiscopic veins lacking	4–6	(3 or) 4–6	3–5	3–5	3–4
Sori position	inframedial to medial	medial	medial	medial to supramedial	medial
Indusium	single	single	single	double	single

### ﻿Chloroplast DNA phylogeny

**Figure 3.**
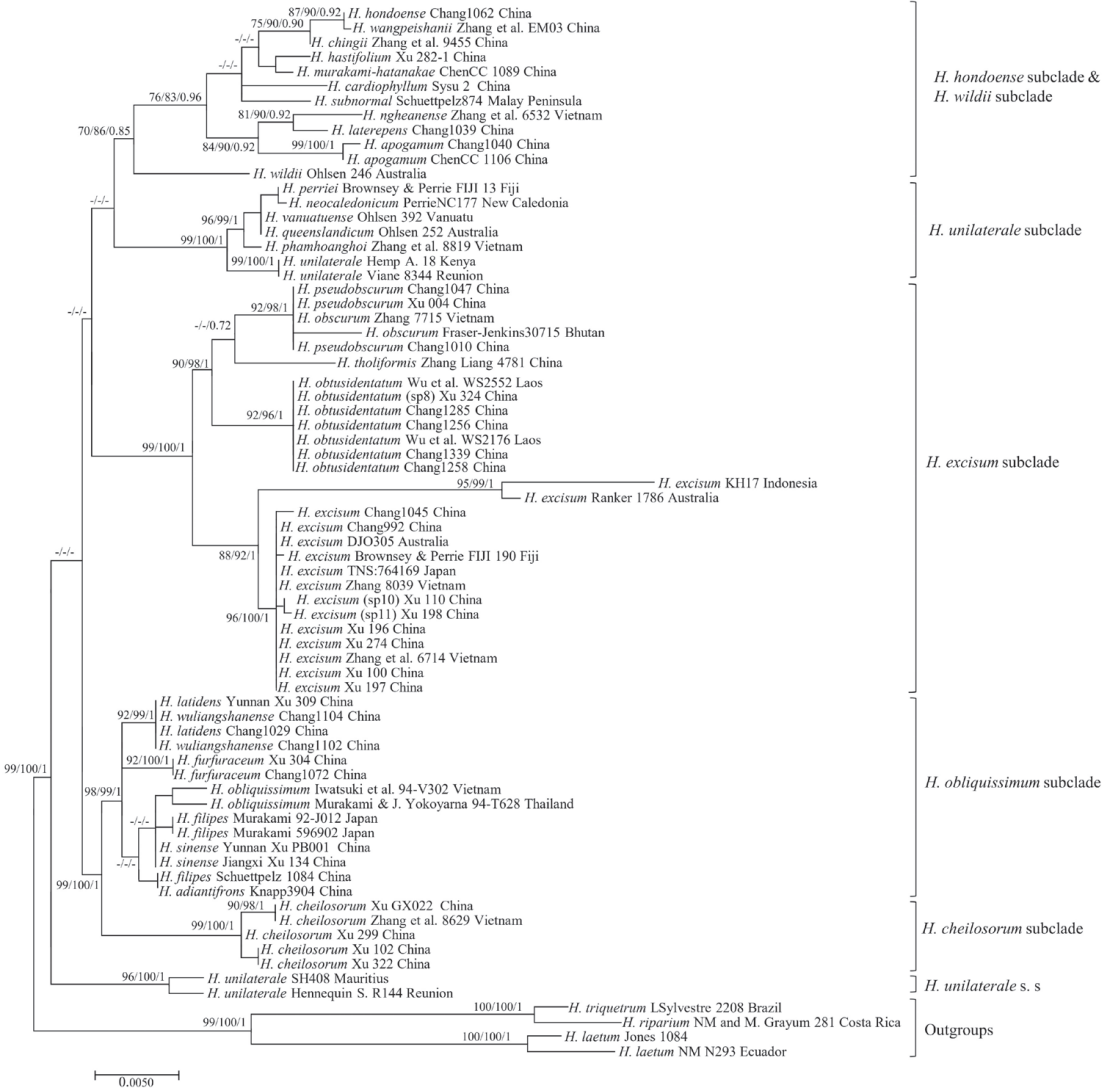
Maximum likelihood phylogeny based on the concatenated plastid DNA sequence dataset. Maximum parsimony and Bayesian analyses recovered identical topologies with respect to the relationships among the main clades of the paleotropical *Hymenasplenium*. For each node, the following values are provided: maximum parsimony bootstrap (%) / maximum likelihood bootstrap (%) / and posterior confidence (p-value). Columns on the right refer to the subclades described in Xu et al. (2018a). Outgroup taxa are shown as a sister to the paleotropical *Hymenasplenium*.

The total length of the concatenated *rbcL*, *rps4-trnS*, and *trnH-psbA* alignment was 2527 bp. The alignment contained 138 variable characters, 98 of which were parsimony informative. The three phylogenetic analyses, MP, ML, and BI, of the combined chloroplast dataset recovered similar topologies (Fig. 3). No significant conflict (bootstrap value > 70%) was detected among the topologies obtained via the separate phylogenetic analyses.

The *H.excisum* subclade recovered in paleotropical *Hymenasplenium* included several well-supported lineages but represented only four described species (Fig. 3). One lineage (BS = 92%, BS = 98%, BI p = 1) was comprised of accessions identified as *H.obscurum* and *H.pseudobscurum*. This lineage formed a separate clade with *H.tholiformis*, but lacked support. Another lineage (BS = 92%, BS = 96%, BI p = 1) was comprised of accessions of the new taxon in this study including collections identified as a proposed new species (sp8, Zhang Liang 4781) and *H.obscurum* (Wu et al. WS2552; Wu et al. WS2176) in former studies (Xu et al. 2018a; Qiu et al. 2022). There was one more lineage (BS = 88%, BS = 92%, BI p = 1) that was comprised of several divergent lineages, but included accessions all identified as *H.excisum*.

## ﻿Discussion

In the present study, the *Hymenaspleniumexcisum* subclade was found to be polyphyletic (Fig. 3), as previously suspected (Murakami 1995; Chang et al. 2018; Xu et al. 2018a). Except for described species, such as *H.excisum*, *H.obscurum*, *H.pseudobscurum*, and *H.tholiformis*, undescribed species are supposed to exist in this subclade (Xu et al. 2018a). The new taxon presented in this study referred to one of the four numbered species (sp8–sp11) in the *H.excisum* subclade proposed by Xu et al. (2018a) in a phylogenetic study of *Hymenasplenium*. The founding of this new species is consistent with the results of the previous studies that cryptic speciation exists and reinforces that urgent study is necessary for species diversity and delimitation within *Hymenasplenium* (Chang et al. 2018; Xu et al. 2018a).

Phylogenetically, the monophyly of *H.obtusidentatum* is strongly supported by the chloroplast sequences analyses (Fig. 3). Morphologically, the distinction of *H.obtusidentatum* is also well reflected. The gross morphology of *H.obtusidentatum* is similar to *H.excisum*, with regard to the laminae and pinna shape, as well as sori arrangement, but differs in the truncate lamina base, not shiny and thinner stipe with color of reddish brown to dark brown, and blunt or rounded pinna marginal teeth (Table 1; Figs 1, 2). Within the *H.excisum* subclade, *H.obtusidentatum* can also be distinguished from *H.obscurum* and *H.pseudobscurum* by not having dull green to grayish green stipe and double indusium. Another species, *H.bivalvatum* (B. K. Nayar & Geevarghese) Viane, was reported to be morphologically similar to *H.pseudobscurum* which also has double indusium but with shiny and dark purple to black stipe, but the occurrence of this taxon in China still needs to be verified (Lin and Viane 2013). The other recently published new species, *H.tholiformis*, has also shiny and dark purple to black stipe but with single indusium. *Hymenaspleniumobtusidentatum* can also be easily distinguished from these two species by having reddish brown to dark brown but not shiny stipe. The distribution of this newly described taxon is currently known in south and southwestern China, and extending to adjacent areas such as Laos, commonly growing together with *H.excisum* on slopes near streams in half-shaded forests.

Though *H.obtusidentatum* is distributed together with *H.excisum* and is morphologically similar to *H.excisum*, this new taxon was found to be closely related to *H.obscurum* and *H.pseudobscurum* in the chloroplast phylogenetic analyses. Different ploidy levels, including diploid, tetraploid, and even hexaploid, are found in *H.excisum*, *H.obscurum* and *H.pseudobscurum*. Besides, reticulations and allopolyploidizations were assumed to occur in *Hymenasplenium* (Chang et al. 2018; Zhang et al. 2021). Thus, the relationships between the new taxon and the remaining species in the *H.excisum* subclade are worth further research. Future studies of multiple biparentally inherited nuclear DNA markers to enhance our ability to trace the evolutionary history of these ferns using statistically consistent methodology, such as coalescence analyses, are warranted.

### ﻿Taxonomic treatment

#### ﻿ Hymenasplenium obtusidentatum

Taxon classificationPlantaePolypodialesAspleniaceae

Y.Fen Chang & G.Cheng Zhang sp. nov.

0971BC2A-F87F-5E5F-958C-66D3A50BB0C8

urn:lsid:ipni.org:names:77306994-1

##### Type.

China. Yunnan Province, Xishuangbanna, Menghai: Chang1258. 2019. (holotype, HITBC; isotype, KUN) (Figs 2, 3).

##### Description.

Plants 25–40 cm tall. Rhizomes long creeping, 2–4 mm in diameter, apex scaly; scales brown, lanceolate to triangular, entire. Fronds remote, up to 6 mm apart, grayish green when dry, herbaceous; stipe reddish brown to dark brown, 10–20 cm long, base ca. 1–1.5 mm in diam., subglabrous, base with sparse scales similar to those on rhizome; lamina one-pinnate, narrowly triangular to triangular, 15–30 × 8–15 cm, base truncate and reduced, apex acuminate to caudate; rachis reddish brown to dark brown, subglabrous; pinnae 15–21 pairs, trapeziform to falcate, basal pinnae nearly opposite, middle pinnae 3.5–8 × 1–1.8 cm, dimidiate, apex obtuse to acute, base asymmetrical, acroscopic side truncate and often almost parallel to rachis, basiscopic side of basal pinnae excavate, in middle pinnae narrowly cuneate and entire, acroscopic margin serrate, teeth blunt or rounded; pinnae spreading to ascending. Veins forking and terminating in marginal teeth, basiscopic side with 4–6 veins lacking. Sori inframedial to medial, linear, 3–5 mm, indusia persistent, pale brown, membranous, entire, opening toward the costa. Spores elliptic to reniform, perispore fimbriate-alate, 38–43 μm in diam.; 64 spores per sporangium.

##### Diagnosis.

*Hymenaspleniumobtusidentatum* is similar to *H.excisum*. However, *H.obtusidentatum* has a thinner rhizome and stipe, not shiny and reddish brown to dark brown stipe and rachis, blunt or rounded pinna marginal teeth, and truncate lamina base.

##### Distribution and habitat.

*Hymenaspleniumobtusidentatum* is currently known to coexist with *H.excisum* in south and southwestern China, and adjacent areas. It occurs in soil or on rocks near streams in half-shaded forests at alt. 1000–1500 m.

##### Additional specimens examined.

China. Yunnan Province, Menghai County, 2019; Chang1256, Chang1260; Chang1261, Chang1262; Chang1339 (HITBC); Pu’er City, Pu’er Sun River National Forest Park, 2018; Xu 324 (SYS). Hainan Province, Ledong County, 2019; Chang1284, Chang1285; Chang1286 (HITBC).

## Supplementary Material

XML Treatment for Hymenasplenium obtusidentatum

## ﻿Appendix 1

**Voucher specimens and GenBank accession numbers for the DNA sequences used in this study. Information is presented in the following order: species, voucher, locality, GenBank numbers for *rbcL* , *rps4* & *rps4-trnS* , *trnH* - *psbA* . “*” represents the newly published sequences in this study; “–” represents no data. Herbaria acronyms follow Index Herbariorum (Thiers 2016).**

***Hymenaspleniumadiantifrons*** (Hayata) Viane & S. Y. Dong; Knapp 3904 (P); Taiwan, China; –, MH065323, –. ***H.apogamum*** (N. Murak. & Hatan.) N. Murak. & Hatan.; Chang1040 (HITBC); xishuangbanna, Yunnan, China; MH884808, MH884830, MH884838. ***H.apogamum***; ChenCC1106 (HITBC); Taiwan, China; MH884813, MH884832, MH884839. ***H.cardiophyllum*** (Hance) Nakaike; Sysu 2; Cult. in Sun-yat Sen University; MH065387, MH065306, –. ***H.cheilosorum*** (Kunze ex Mettenius) Tagawa; Xu 102 (SYS); Hainan, China; MH065385, MH065346, –. ***H.cheilosorum***; Xu 299 (SYS); Guangxi, China; MH065402, MH065350, –. ***H.cheilosorum***; Xu 322 (SYS); Yunnan, China; MH065405, MH065352, –. ***H.cheilosorum***; Xu GX022 (SYS); Guangxi, China; MH065381, MH065343, –. ***H.cheilosorum***; Zhang et al. 8629 (CDBI, MO, PHH); Lam Dong, Vietnam; MH065415, MH065357, –. ***H.chingii*** K. W. Xu, Li Bing Zhang & W. B. Liao; Zhang et al. 9455 (CDBI); Guizhou, China; MH065430, MH065333, –. ***H.excisum*** (C. Presl) S. Lindsay; Brownsey & Perrie FIJI 190 (WELT); Fiji; KP774884, KP851882, –. ***H.excisum***; Chang1045 (HITBC); Yunnan, China; OP375802*, OP375808*, –. ***H.excisum***; Chang992 (HITBC); Yunnan, China; OP375801*, OP375807*, –. ***H.excisum***; DJ0305; Kaaruu Creek, Queensland, Australia; KP774930, –, KP851883. ***H.excisum***; Xu 274 (SYS); Guangdong, China; MH065389, –, –. ***H.excisum***; KH17; Indonesia; GU586828, –, –. ***H.excisum***; Ranker 1786 (COLO); Hawaii; AY549728, –, –. ***H.excisum***; TNS:764169; Japan; AB574888, –, –. ***H.excisum***; Xu 100 (SYS); Hainan, China; MH065383, MH065344, –. ***H.excisum***; Xu 110 (SYS); Hainan, China; MH065384, MH065345, –. ***H.excisum***; Xu 198 (SYS); Hainan, China; MH065394, MH065341, –. ***H.excisum***; Xu 196 (SYS); Hainan, China; MH065390, –, –. ***H.excisum***; Xu 197 (SYS); Hainan, China; MH065391, –, –. ***H.excisum***; Zhang et al. 6714 (CDBI, MO, VNMN); Bac Kan, Vietnam; MH065436, MH065375, –. ***H.excisum***; Zhang et al. 8039 (CDBI, MO, VNMN); Quang Nam, Vietnam; MH065419, MH065361, –. ***H.filipes*** (Copeland) Sugimoto, Murakami 596902 (TI); Kagoshima, Japan; U30605, –, –. ***H.filipes***; Murakami 92-J012; Kagoshima, Japan; AB016176, –, –. ***H.filipes***; Schuettpelz_1084 (HITBC); Taiwan, China; MW194197, –, –. ***H.furfuraceum*** (Ching) Viane & S. Y. Dong; Chang1072 (HITBC); Yunnan, China; MW194198, MW194221, MW194182. ***H.furfuraceum***; Xu 304 (SYS); Yunnan, China; MH065406, MH065353, –. ***H.hastifolium*** Ke Wang Xu, Li Bing Zhang &W. B. Liao; Xu 282-1 (SYS); Guangxi, China; MH065398, MH065313, –. ***H.hondoense*** (N. Murak. & Hatan.) Nakaike; Chang1062 (HITBC); zhaotong,Yunnan, China; MH884814, MH884833, MH884840. ***H.laetum*** (Sw.) L. Regalado & Prada; Jones 1084 (TUR); KJ628715, –, –. ***H.laetum***; NM N293; Ecuador; AB014707, –, –. ***H.laterepens*** N. Murak. & X. Cheng ex Y.Fen Chang & K. Hori; Chang1039 (HITBC); xishuangbanna, Yunnan, China; MH884807, MH884829; MH884837. ***H.latidens*** (Ching) Viane & S. Y. Dong; Chang1029 (HITBC); Yunnan, China; MW194204, MW194219, MW194180. ***H.latidens*** (Ching) Viane & S. Y. Dong; Xu 309 (SYS); Yunnan, China; MH065407, MH065318, –. ***H.murakami-hatanakae*** Nakaike; ChenCC1089 (HITBC); Taiwan, China; MH884823, –, –. ***H.neocaledonicum*** Li Bing Zhang & K. W. Xu; PerrieNC177 (WELT); New Caledonia; KP774896, KP851878, –. ***H.ngheanense*** Li Bing Zhang, K. W. Xu & N. T. Lu; Zhang et al. 6532 (CDBI, MO, VNMN); Phu Tho, Vietnam; MH065426, MH065331, –. ***H.obliquissimum*** (Hayata) Sugimoto; Iwatsuki et al. 94-V302; Hoang Lien Son, Vietnam; AB016187, –, –. ***H.obliquissimum***; Murakami & J. Yokoyarna 94-T628; Chiang Mai, Thailand; AB016178, –, –. ***H.obscurum*** (Blume) Tagawa; Fraser-Jenkins30715; Bhutan; MH884826, MH884834, –. ***H.obscurum***; Zhang et al. 7715 (CDBI, MO, VNMN); Thanh Hoa, Vietnam; MH065411, MH065354, –. ***H.obscurum***; Wu et al. WS2176 (MO); Xiangkhoang, Laos; ON85986, ON859875, –. ***H.obscurum***; Wu et al. WS2552 (MO); Louangphrabang, Laos; ON859870, ON859876, –. ***H.obtusidentatum*** Y.Fen Chang & G.Cheng Zhang; Chang1256 (HITBC); Yunnan, China; OP375803*, OP375809*, OP375813*. ***H.obtusidentatum***; Chang1258 (HITBC); Yunnan, China; OP375804*, OP375810*, OP375814*. ***H.obtusidentatum***; Chang1285 (HITBC); Hainan, China; OP375805*, OP375811*, OP375815*. ***H.obtusidentatum***; Chang1339 (HITBC); Yunnan, China; OP375806*, OP375812*, OP375816*. ***H.perriei*** Li Bing Zhang & K. W. Xu; Brownsey & Perrie FIJI 13 (WELT); Fiji; KP774885, KP851880, –. ***H.phamhoanghoi*** Li Bing Zhang, K. W. Xu & T. T. Luong; Zhang et al. 8819 (CDBI, MO, PHH); Khanh Hoa, Vietnam; MH065432, MH065334, –. ***H.pseudobscurum*** Viane; Chang1010 (HITBC); Yunnan, China; MH884827, MH884835, MH884841. ***H.pseudobscurum***; Chang1047 (HITBC); Yunnan, China; MH884828, MH884836, MH884842. ***H.pseudobscurum***; Xu 004 (SYS); Hong Kong, China; MH065380, MH065342, –. ***H.queenslandicum*** Li Bing Zhang & K. W. Xu; Ohlsen 252 (MELU); Queensland, Australia; KP774849, KP851879, –. ***H.riparium*** (Liebm.) L. Regalado & Prada; NM and M. Grayum 281; Virgen del Socorro, Costa Rica; AB014708, –, –. ***H.sinense*** K. W. Xu, Li Bing Zhang & W. B. Liao; Xu 134 (SYS); Jiangxi, China; MH065388, MH065348, –. ***H.sinense***; Xu PB001 (SYS); Yunnan, China; MH065386, MH065347, –. ***H. sp8***; Xu 324 (SYS); Yunnan, China; MH065404, MH065351, –. ***H.subnormale*** (Copel.) Nakaike; Schuettpelz 874; Malay Peninsula; MH884824, –, –. ***H.tholiformis*** Liang Zhang, K. W. Xu & W. B. Ju; Zhang Liang 4781 (KUN); Tibet, China; ON859868, –, ON859874. ***H.triquetrum*** (N.Murak. & R.C.Moran) L.Regalado & Prada; L.Sylvestre 2208 (RB); Brazil; KT329398, −, –. ***H.unilaterale*** (Lam.) Hayata; Hemp A. 18 (BM); Kenya; AF240652, –, –. ***H.unilaterale***; Hennequin S. R144 (BM, REU); Reunion; KF992497, –, –. ***H.unilaterale***; RV8344; Reunion; GU929873, –, –. ***H.unilaterale***; SH408; Mauritius; MH884825, –, –. ***H.vanuatuense*** Li Bing Zhang & K. W. Xu; Ohlsen 392 (MELU); Tanna, Vanuatu; KP774898, KP85188, –. ***H.wangpeishanii*** Li Bing Zhang & K. W. Xu; Zhang et al. EM03 (CDBI); Sichuan, China; MH065410, MH065333, –. ***H.wildii*** (F. M. Bailey) D. Ohlsen; DJO246 (MELU); Queensland, Australia; KP774927, KP851877, –. ***H.wuliangshanense*** (Ching) Viane & S. Y. Dong; Chang1102 (HITBC); Yunnan, China; MW194208, MW194224, MW194184. ***H.wuliangshanense***; Chang1104 (HITBC); Yunnan, China; MW194209, MW194225, MW194185.
